# Addressing the Gap of Nutrition in Medical Education: Experiences and Expectations of Medical Students and Residents in France and the United States

**DOI:** 10.3390/nu15245054

**Published:** 2023-12-09

**Authors:** Solenn Thircuir, Nancy N. Chen, Kristine A. Madsen

**Affiliations:** 1 ESA Business School, Beirut B.P. 113-7318, Lebanon; solenn.thircuir@live.fr; 2 Department of Anthropology, University of California, Santa Cruz, CA 95064, USA; 3 Berkeley School of Public Health, University of California, Berkeley, CA 94710-7360, USA; madsenk@berkeley.edu

**Keywords:** nutrition education, medical curriculum, medical training, culinary medicine

## Abstract

Distinct pedagogical approaches within medical curricula in France and in the U.S. reflect a growing recognition of the importance of nutrition to address major public health challenges. However, recent generations of medical students have expressed mixed opinions regarding nutrition education. What pedagogical approach may improve nutrition education? Despite different medical systems, students from both France and the U.S. share similar concerns and expectations, that nutrition knowledge must be embedded in the curriculum and must be engaging. Hands-on, system-based, epistemological, and multidisciplinary approaches need better articulation to forge a robust medical curriculum. In the rapidly changing contexts of medicine and public awareness, social science research may facilitate recommendations for improved nutrition education.

## 1. Introduction

Nutrition-related disorders, such as overweight, obesity, undernutrition, or deficiency, are a major public health problem. According to the World Health Organization, chronic diseases are responsible for 8 out of 10 deaths, and heart disease remains the number one killer. In France, one third of deaths occurring before 65 years of age is related to modifiable factors such as diet, malnutrition, and sedentary lifestyle. In the U.S., disease-related and other forms of malnutrition remain a serious issue affecting more than 30% of hospitalized patients [1], while poor diet is the leading contributor to mortality [2]. Nutrition education encompasses a spectrum of institutions that determine medical training, hospital structures, and political decisions. The challenges of nutrition education are not solely limited to public policy, as ethical questions also shape medical training. Poor diet is a prevalent social issue and health problem that concerns the practice of all physicians across all specialties.

Nutrition appears to be partially, if not totally, absent from medical education programs, not only in the U.S. but also in Europe according to a recent study published in The Lancet Planetary Health [3]. In the U.S., 86% of physicians report they feel unqualified to offer nutritional advice to patients [4]. Persistent, widespread poor dietary patterns, despite robust public health policies in both countries, impels us to evaluate the relevance and adequacy of nutrition education in medical training [5,6]. According to a qualitative study conducted with US medical students, nutrition education is perceived to be inadequate in the medical curriculum due to personal, interpersonal, and environmental barriers [7], which is also the case in Europe. This impediment led to the first White House Conference on Hunger, Nutrition, and Health on 28 September 2022 hosted by the Biden–Harris administration, which was set up to highlight the need for more nutrition education. This new challenge followed U.S. House of Representatives Resolution 1118 which called for nutrition education for all medical students and physicians as a public health priority. Earlier in France, the Ministry of Health and Prevention, in March 2019, deemed food, nutrition, and physical activity state priorities, establishing clear goals such as decreasing obesity rates by 2023.

Once learned, new knowledge or skills are incorporated into professional practice. However, this process is not always straightforward or easily implemented. The acquisition of nutrition knowledge takes place in complex contexts, requiring medical students and residents to integrate various knowledge resources. Understanding medical students and residents’ conceptions of nutrition pedagogy may shed light on how medical schools may frame such work for future physicians to fully engage their patients. This article addresses the following issues: What are the different approaches to nutrition education in France and in the U.S.? How do medical students and residents experience their nutrition medical training in both systems? How might medical schools align their curriculum to help future physicians engage with nutrition knowledge? What pedagogical approach may improve nutrition education for future generations of medical practitioners?

We hypothesize that medical students and residents’ perspectives on nutrition are framed by medical schools’ approaches to nutrition education which pose key opportunities to revise the medical curriculum. This article examines how medical students approach nutrition knowledge to understand the disconnect between student knowledge developed outside the curriculum and the information gained through medical training. For decades, research on medical pedagogies tended to focus on the role of the learner. Most medical curricula emphasized the memorization of human anatomy, but they rarely engaged systemic analysis for students to consider how patients might exist within complex systems, such as food and healthcare systems. Moreover, nutrition has a huge impact on long-term wellbeing and patient outcomes. Ethnographic research as well as pedagogical analysis may identify effective forms of nutrition education. Since nutrition knowledge is insufficiently implemented in medical curricula to meet public health challenges of chronic diseases, we analyze and propose effective teaching approaches.

## 2. Materials and Methods

The Committee for the Protection of Human Subjects at the University of California, Berkeley, approved this research in March 2022 based on the submitted surveys and interview questions drafted by the first author and reviewed by the second and third authors. To fill the aforementioned research gap, 30 qualitative, semi-structured interviews with medical students and residents from a dozen medical schools across the two nations were conducted in the years 2022 and 2023 [please refer to acknowledgements]. Seven interviews involved residents. The interviewees were between 19 and 36 years of age, and 19 of them were women and 11 were men. At the beginning of each interview, and before starting the recording, the interviewees were systematically asked to give their consent. Interviewees were asked open-ended questions about how they value the importance of nutrition, the way they have been taught nutrition, their perception of and satisfaction with the curriculum as well as suggestions for improvement. The interviews were conducted over the phone or via video calls. The interviews lasted between approximately half an hour and one and a half hours each; most of them lasted around one hour. All interviews were carried out in French and English, fully transcribed, and translated into English.

Access to the interviewees was gained by “snowball sampling” [8] with medical instructors acting as intermediaries. We selected the interviewees based on their involvement in the medical curriculum. Throughout the project, we first interviewed medical students and residents in France and then in the U.S. In addition, one of the authors conducted an on-site ethnography and attended a nutrition class in a medical school in the U.S., where she got a tour of the facilities and conducted an observation of a morning class. Lead author Thircuir engaged in participant observation in culinary medicine courses and attended the Teaching Kitchen 2022 Conference and the ACGME summit regarding the next steps to incorporate nutrition education into the medical curriculum in the U.S. The knowledge developed through this ethnography inspired our analysis and the development of this article, which is, however, mainly based on interview data.

The interviews were inductively coded following the grounded theory approach [9]. Thus, we developed our analysis from the empirical material which was, step by step, grouped into categories of higher abstraction. We continued this process throughout the whole data collection period.

Multiple health professionals, researchers, experts as well as policymakers illuminated epistemological reflections and the need for improvement. Building on these insights, this article examines how medical education systems address the significance of nutrition to recommend future multidisciplinary training programs, core competencies, and pedagogical approaches and meet future health professionals’ expectations. To assess existing research, we commenced with a search of the relevant literature on medical education and nutrition training in both countries. From the initial review, we selected specific references that offered structural insights on the intersections between the medical curriculum and nutrition in medical schools as well as the ongoing gaps in nutrition training.

As detailed below, we first explore the socio-historical perspectives on the status and articulation of nutrition knowledge within medical systems. We then reflect on the medical students and residents’ experiences of their education and why they share similar views on the gap in nutritional training. Finally, we examine ways to improve programming and recommend future multidisciplinary and wellness care teams within medical schools.

## 3. Results

### 3.1. Historical Contexts and Acknowledgement of Nutrition within Medical Systems

Nutrition as a science was constituted during the 20th century from the biochemical definition of the concept of nutrients and the various interactions with sciences such as genetics, immunology, sociology, and pharmacology [10], mainly based on biological mechanisms. Social scientists and historians of food identify how, at key moments of nutrition research, varying definitions of nutrition (especially good nutrition) have shaped dietary recommendations as well as eating habits [11,12]. Twentieth-century perspectives led to the current framing of nutrition knowledge and to various nutritional strategies aimed at preventing and developing good eating habits. In 1980, the USDA and HHS collaboratively issued Nutrition and Your Health: Dietary Guidelines for Americans in the U.S., while in France, in 2001, the National Nutrition and Health Plan (PNNS) was created. These initiatives have provided guidelines and aimed, within the framework of public health at the national level, for a better state of health for U.S. and French citizens. The enhanced understanding of metabolism and the ability to study nutrients emphasized the link between nutrition and health, which further demonstrated the need for a nutrition curriculum in medicine [13]. 

In the U.S., there has been a constant call for and effort to improve the nutrition education of physicians since the mid-20th century. In 1963, the American Council on Foods and Nutrition noted, “There is an urgent need to define the responsibilities and challenges of medical schools in the teaching of nutrition. Modern research has shown the importance of nutritional factors in the pathogenesis and therapy of disease and the importance of nutrition in conditions of physiologic stress” [14]. In France, there were also advances to improve this situation. In 1988, with the creation of a sub-section of nutrition in the National Council of Universities (CNU), nutrition was recognized as a discipline to be taught in the same way as anatomy or physiology. Moreover, nutrition instructors created the College des Enseignants en Nutrition (College of Nutrition Educators) with objectives to promote nutrition education in medical schools, define pedagogical objectives for undergraduate and postgraduate courses, and serve as a resource to national authorities. These initiatives and efforts led to nutrition being implemented into the medical curriculum in some medical schools in both countries. 

Despite these efforts, there were shortcomings in the implementation of nutrition education in both countries. A survey conducted by the Nutrition in Medicine team at the University of North Carolina (Nutrition in Medicine is a project which aims to develop knowledge and skills about nutrition for future physicians, http://nutritioninmedicine.org/, accessed on 16 August 2023) found that, since 2000, most responding medical schools failed to provide the minimum 25 h of nutrition education recommended by the National Academy of Sciences [15]. In Europe, the same survey was conducted in 2014 and generated similar results. These studies not only reflect a lack of interest in this field, but also the challenges of implementing nutrition as part of the core curriculum for medical schools. Its alignment with other disciplines was an obstacle to facilitating the place of nutrition in medical curricula. Other challenges were the competition with other medical disciplines and the vast array of knowledge physicians are expected to gain during training. To meet these challenges, an expanded role for consulting dietitians in patient care could be a possible alternative when nutrition education is not feasible. Medical student respondents in both countries welcomed the intervention of dietitians and nutritionists which we address in our findings. 

Nutrition has never been systematically recognized as a medical discipline. The American Society for Nutritional Science defined core nutrition knowledge as overlapping with other disciplines: chemistry, biochemistry, molecular biology, epidemiology, food science, genetics, immunology; these also interface with other disciplines such as oncology, pharmacology, neurobiology and involve humanities and social sciences. Considering nutrition from these historical and systemic perspectives helps to identify epistemological issues that determine medical training. 

### 3.2. Development of Nutritional Programs within Medical Training

To align with other medical disciplines and the biomedical approach of health, most medical schools that teach nutrition do so through the physiology and biochemistry of nutrients. The objective of nutrition courses is the acquisition of basic scientific knowledge, which is essential for the subsequent mastery of knowledge and medical practice. The basic understanding of nutrition focuses on diet for optimal growth, proper metabolism, the maintenance of tissues, and the avoidance or recovery from certain disease states. Nutrition education includes the study of components such as fat, protein, vitamins, minerals, trace elements, water, fiber, and calories which aligns with the structure of the overall medical curriculum. 

We comparatively assessed medical education by structure and for any inclusion of nutrition knowledge or training. We included three French medical schools and nine American medical schools, and the selection criteria were based on snowball sampling as well as classic ethnographic methodology to understand how medical students encountered and experienced the medical curriculum and whether or not these curricula had a nutrition component. The years covered, based on this approach, reflect the past six years. In France, medical studies are divided into three cycles, and the first three-year cycle of “general training” ends with a diploma of general training in medical sciences. Nutrition knowledge is evaluated during this first cycle. French students usually enter medical school right after high school, while American students attend college prior to medical school. In the U.S., the pre-clinical years, typically the first and second years at medical school, consist of courses in basic sciences and discipline-specific instruction. In both systems, careful guidance by instructors initiates students into traditional concepts of medicine (Table 1). 

Not all medical schools in either country have implemented nutrition into their curriculum. French medical schools include biochemical aspects of nutrition incorporated into the endocrinology curriculum, but do not include training on how to provide concrete dietary advice to patients. There is no unified approach in US medical schools. The diversity of approaches as well as medical students and residents’ varied experiences of nutrition pedagogy offers significant opportunities to reflect on their training as well as the competencies needed. Regarding the percentage of medical students that receive nutrition education in the U.S., in 2015, only 26 out of 105 medical schools offered a specific nutrition course for future physicians [16]. The average training time was 19.6 h, the majority of which was delivered in basic science courses, i.e., biochemistry, physiology, and pathophysiology, during preclinical training. Among the 37 medical schools in France, all of them offer compulsory nutritional classes except 3, according to a Professor of Nutrition at the University of Paris interviewed for this research, because of the lack of qualified lecturers and professors in the field.

Compulsory nutrition education can impact health outcomes. A US study evaluated the significance of nutritional counseling on the quality of care [17]. It states that it is most effective when healthcare providers meet patients where they are and provide a tailored, realistic approach to healthy weight loss. It adds that motivational interviewing techniques can be used to achieve these goals. In France, a study has shown that insufficient medical training is often experienced negatively by patients, with repercussions on the interest and effectiveness of practitioners [18].

The College des Enseignants en Nutrition agreed that nutrition would be incorporated within endocrinology, an implicit suggestion that medical nutrition on its own is not considered a science but is an integral part of physiology, biochemistry, cell or molecular biology. This approach is the most common in both countries. Nutrition is compulsory in France, as it is evaluated in board exams; medical students study for the subject, but are not necessarily formally taught. In the U.S., nutrition education may be required or not depending on the individual medical school’s position. In both countries, nutrition has mainly been taught in stand-alone modules or in a fragmented manner in various disciplines but is rarely integrated across the curriculum.

These epistemological difficulties led to different approaches that have been recently developed in the U.S. Over the past decade, culinary medicine (CM) programs developed within different medical schools aimed at teaching nutrition as a student-centered approach focusing on knowledge construction processes and a content-oriented and hands-on transmission approach. CM programs such as Teaching Kitchens and Health Meets Food are programs that have been implemented across the U.S. but not yet in France. CM programs were created to teach the significant role of food choices and nutrition in health and overall wellbeing as well as the curative and preventive aspects of these food choices. CM programs are not limited to medical schools; they often develop collaborations with local communities, hospitals, schools and involve actors from the healthcare system and the food industry. The curriculum helps medical students understand the impact of food on the health of their patients and their own health. CM instruction usually involves a physician and a chef in order to bring the basic science curriculum together with clinical education while incorporating dietary intervention strategies into the practice of medicine. Through hands-on cooking classes, students learn the practical aspects of dietary change. The approach is centered on encouraging a reflection of one’s own food intake by which future physicians will be able to guide their patients to making healthier food choices. CM aims at incorporating dietary intervention strategies into the students’ practice of medicine. Programs such as Teaching Kitchen, Health Meets Food, and other initiatives across the country have participated in highlighting the importance of nutrition education in medicine. By facilitating a complementary teaching–learning environment for nutrition knowledge acquisition, the CM approach enables the integration of food as a medical continuum, rather than treating nutrition and medicine as separate entities [19]. 

The development of distinct approaches to nutrition education in French and American medical schools as well as the consistent efforts for improvement reflect the gradual recognition of the importance of nutrition to address major public health challenges. Below, we share medical students and residents’ experiences of these programs.

### 3.3. Findings from Semi-Structured Interviews with Medical Students and Residents

#### 3.3.1. Preconceptions about Nutrition’s Importance

Participants’ personal experiences outside medical school shaped their understanding and expectations regarding nutrition knowledge and overall medical practice. As a generation raised during the creation and growth of social media as well as navigating the pandemic, many respondents mentioned how they boosted their own immunity through nutritional intake. The nature of personal knowledge that concerns beliefs that an individual has about formal knowledge is what Hofer and Pintrich called “personal epistemology” [20]. Analyzing the interviewees’ understanding of nutrition through this lens enables a consideration of how they engage with information and resources to make sense of nutrition knowledge. These factors also shape their expectations regarding teaching and learning about nutrition with regard to health and disease. In 2008, a survey in France showed that health professionals and dieticians represented only the fourth and sixth source of nutritional information, after written and audiovisual media, the internet, parents and friends (http://www.inpes.sante.fr/Barometres/barometre-sante-2014/index.asp, accessed on 10 June 2023). Interviewees refer to public health issues of how nutrition claims take shape, spread widely, and are taken into account by public authorities.

When asked to define nutrition, interviewees from both countries referred to nutrition as fundamental in the preservation of health and the prevention and healing of disease. Students consider that nutrition impacts mental and physical states. Underlining this is the notion that nutrition is not a distinct and separate concept when it comes to health. Indeed, all interviewees consider that medicine and nutrition are inseparably linked and that the nutritional state is a condition significant to the preservation and restoration of health. This appears to cohere with the World Health Organization’s definition of health as “a state of complete physical, mental and social well-being and not merely the absence of disease or infirmity” (1948), meaning that nutrition is not only inseparable from health, but is also a global approach for human wellbeing. A fifth-year medical student at the University of Lyon affirmed this approach to nutrition, stating that it “has a preventive role and could really help in the health status of the population”. 

Medical students commence medical education with preconceptions about nutrition’s importance in health based on prior experiences. They build upon their experiences and incorporate new information taking a constructivist approach that actively integrates new and old knowledge concerning nutrition. They build their knowledge base in a context of proliferating discourses and expertise. A second-year medical student at the University of Cleveland expressed the need for clarification in updating nutrition knowledge: “There are ways that we can instill the knowledge more longitudinally because it is relevant for many things like immunity, for example. For optimal disease fighting capability, I think there’s a lot of room for instilling more education”. Nutrition knowledge appears to be evolving for the vast majority of interviewees, as a fourth-year medical student at the University of California, Davis, noted, “We should stay up to date on how nutritional guidance continues to change”. These narratives indicate that students and residents’ epistemological belief in the learning process builds upon existing knowledge. The students emphasize the importance of problem solving, interaction, and collaboration. They believe active, self-directed learning efforts to be integral to nutrition knowledge. A first-year resident at the University of Nevada, Las Vegas, attests to having to “seek out the education” himself, since nutrition is not “brought to me”. He stays informed by searching for scientific articles and listening to popular science podcasts on the subject. For a second-year medical student at the University of Nevada, Las Vegas, nutrition knowledge should be an active construction process during medical training: “With our medical education, we have access to a lot of different resources that help us see what certain things that we might need in our own diet”. The student is interested in actively constructed knowledge rather than passively absorbed knowledge. Nutrition instructors should not simply transfer knowledge to students but facilitate students’ active knowledge construction processes to gain an understanding of the subject matter. Discussing a diversity of approaches and understanding their coexistence would make it possible for nutrition courses to be spaces for engaged analysis. A resident at Columbus Nationwide Children’s Hospital relates to the challenges to address the cultural differences in consultation: “I struggle with some cultural differences too. For example, some cultures like their babies chubby that shows that they’ve been loved. Those are things I definitely struggle with when talking about diet with families”. A French resident at the University of Marseille adds that individual relationships with food are unique and also involve taking into account social and cultural factors: “We don’t realize the emotional, cultural, and family aspects that may be involved. And as long as we don’t try to understand it, we can’t change patients’ eating habits”. The respondents expressed that they would strive for an environment that recognizes their needs and offers an approach for expressing, challenging, and sharing ideas through dialogue. The interviewees shared concerns that the nutrition curriculum was not practical, in part because the courses do not provide clear directions for medical practice nor take into account that physicians overwhelmingly feel that patients do not follow lifestyle guidance. A third-year medical student at Loma Linda University mentioned his feeling of dissonance, a gap between the importance, beliefs, and expectations associated with nutrition and the way the subject is implemented into the medical curriculum: “It’s a disconnect from what I do versus what I believe. I would say it’s super important for maintaining good health long, especially long term”. Respondents in both countries shared the common idea of holistic and preventive aspects of nutrition. Yet, nutrition has been defined as fundamentally biological and as a process by which individuals use food to find a nutritional state and maintain an adequate state of health, integrating physiological, social, cultural, and emotional elements. A second-year medical student from the University of Cleveland describes a gap in relation to medical practice during consultations: “There is a disconnect between how and what we’re supposed to say and what we actually do to make changes. There’s a complacency in medicine that people aren’t going to make those changes. So you just give medication […] I was never trained on how to counsel them”.

#### 3.3.2. Clinical Reasoning Creates a Separation between Concrete and Abstract Knowledge

Medical students are taught clinical reasoning based on evidence. Medical decision-making is also based on the rationalization and standardization of care as well as current modes of clinical practice. Indeed, clinical reasoning applied to nutrition creates a separation between concrete and abstract knowledge and its implementation in practice, making students feel either that nutrition is too obvious a concept to be worth learning about, is not related to medicine at all, or that they will not remember what they are being taught. According to a fourth-year medical student at the University of Lyon: “I find that instructors have a tendency to say “okay, we should do this”, but not really to think about “how can I improve this?” Additional training to deepen medical training in nutrition within clinical practices is necessary to ensure a long-term engagement with nutrition knowledge for patients. Interviewees mention the gap between their beliefs and the medical culture. They are aware of the constraints that medical schools face to develop and adapt their curriculum which is based on evidence-based knowledge which might not allow for the integration of other approaches. This tension was identified by the fourth-year medical student at the University of California, Davis: “Eating and lifestyle is personal. Med schools probably won’t teach [these topics] unless there’s a lot of evidence”. Nutrition within medical schools is presented as a technical and neutral way of designating a biochemical approach to nutrients which connotes a legitimate scientific approach. Some French medical students interviewed were not aware of the existence of nutrition classes, or some decided not to attend them even if they were mandatory. A fourth-year resident at the University of Marseille stated that she cannot remember for sure if she had nutrition classes. These narratives are not to be confused with a lack of interest in nutrition. On the contrary, the interviews suggest that medical students and residents who value their learning efforts positively feel less dependent on the expertise of the instructor. Medical students and residents are confronted with a difficult contradiction: the insufficiency of their training and the lack of recognition of nutrition as a specialty while acknowledging the importance of nutrition in the maintenance of health. This is not only a matter of knowledge acquisition, but also of the competencies at stake. Students on both sides are concerned about the need to gain deeper knowledge. The lack of interest is a consequence of knowledge being perceived as too abstract to be incorporated into the medical practice.

#### 3.3.3. Perceived Challenges to Incorporating Preventive and Holistic Approaches of Nutrition into the Curriculum

Approaches to medical practice as taught in their curriculum led medical students and residents to perceive barriers to providing dietary advice as well as a lack of confidence in the field. They perceived medical schools to lack interest in nutrition, as a third-year medical student at Loma Linda University summarized, stating that “It’s undervalued in medical schools in general”, coming to the conclusion that nutrition is “left to other professionals or for learning on our own”. Medical students and residents’ awareness of the difficulties in accessing nutrition education that aligns with their prior knowledge and other sources lead them to put aside expectations in order to succeed in their medical training. They will have to—at least temporarily—adhere to the biochemical approach of nutrition and its pedagogical approach in order to advance. The interviewees’ expressions of disconnect between their prior knowledge and their understanding of nutrition from a biochemical lens tend to promote certain sources of knowledge as being illegitimate from a medical perspective. In both countries, medical students assert that the medical schools should take on the responsibility to adapt their curriculum in order to address the importance of nutrition. Despite the nuances of the respondents’ responses, many find that holistic and preventive approaches to nutrition are not incorporated into the curriculum. Nonetheless, the respondents do not feel entirely determined by their medical training and still believe that they will continue to incorporate other sources of information in the long term. As a fourth-year medical student at the University of California, Davis aptly summarized: “I think taking a bigger lens to approach nutritional fads because those will like come and go throughout our careers”.

#### 3.3.4. A Feeling of Disconnection and Distrust of Medical Schools

Despite some interviewees’ awareness of the medical approach and the legitimate difficulty for medical schools to implement nutrition education, such gaps may feel like a resistance and deferral of medical schools’ responsibility especially when nutrition appears as a major public health concern. As a fourth-year resident at Loma Linda University noted: “The top risk factors, the top things that kill patients in their country [are] dietary-risks; why are we not addressing that [nutrition] more heavily?” Even while nutritional policies promote health values to address poor nutrition and diet-related disease as top priorities, we note an inconsistency within existing normative frameworks of training and medical practice. Such disconnections consequently produce a distrust towards medical schools as well as their scientific authority. Medical students and residents are concerned about the quality of the information available to them. The attitudes and skills of instructors in nutrition have often been questioned by the interviewees in France and the U.S. In the U.S., a study indicated that a physician’s knowledge of nutrition may even be inferior to that of the patient on certain topics [21]. In the rapidly changing contexts of medical practice and public awareness, it is critical to examine ways to improve programming and develop recommendations to adapt the curriculum to new health challenges. 

## 4. Discussion: Recommendations for Future Nutrition Training within Medical Schools

Despite differences in medical education systems between France and the US, we found that medical trainees are aware of the gap between what they are being taught and their expectations. The mismatch between these elements leads to an effort on the part of the individual to bridge that gap [22]. They want nutrition to incorporate approaches that provide hands-on experiences and a system-based perspective while promoting critical thinking and referencing the social and cultural aspects of nutrition.

With CM classes in the U.S., medical students and residents expressed more satisfaction due to the concrete and hands-on practice. Despite satisfaction with CM classes, there were, however, still discrepancies that were raised. Some students explained that they did not find the information sufficiently relevant to prepare them to advise patients, or that the timing of the course did not align with lunch or dinner time, making them less motivated to cook. The cultural and social aspects of food intake were not always addressed. Overall, the interviewees’ knowledge, food practice, personal experience, collective reflections, and broader public health issues need to be considered.

Nutrition knowledge is not solely determined by medical or scientific knowledge, but is shaped by different discourses and experiences. The uncertainties surrounding nutrition knowledge and public health awareness regarding diet-related diseases reflect the inherent tension in the status of nutrition from a clinical versus holistic perspective. At present, medical education is organized by discipline: gastroenterology, endocrinology, pneumology, cardiology, etc. Connecting nutrition education with systemic knowledge is central to acknowledge the distinctions between treatment and care. The full participation of different disciplines would enable students to approach nutrition across specialties and establish their identity as physicians. Medical authorities and health institutions, while concerned about the magnitude of this problem, continue to expect physicians to assume this responsibility without fully providing adequate curricular material. For example, the PNNS set up by the Ministry of Health in France, which aims to improve the health status of the population by acting on nutrition, does not provide for any improvement in the training of physicians. Medical students and residents’ adherence to professional ethics does not solve their incompetence regarding nutrition. Nutrition pedagogies within medical school curricula also need to engage with the diversity and values of food in society. According to the students interviewed in both countries, a key challenge during consultation is the ability to advise patients while taking into account cultural differences and expectations. Developing a patient-centered approach regarding nutrition needs to incorporate nutrition counseling which takes into account the impact of cultural factors and the diversity of personal perceptions. The students state that there needs to be recognition of the disparities in foodways and nutrition and therefore point out how nutrition is also a matter of social justice.

## 5. Conclusions

This study examines the challenges and opportunities for nutrition education in medical school training and is based on assessments of historical contexts, semi-structured interviews, and a selective literature review surveying previous studies on the topic in social sciences and public health. Our findings indicate that hands-on, system-based, epistemological, and multidisciplinary approaches may help forge a robust nutrition education within the medical curriculum. Nutrition knowledge and culinary medicine can be effective as part of overall interdisciplinary engagements to address current gaps in medical education.

## Figures and Tables

**Table 1:** Nutrition in medical system curricula between France and the US.

	France	United States
Structure	Pre-clinical years (1–3 years): general training in medical sciences. Clinical years (3 years): pathological processes, treatment and prevention, organization of healthcare systems, evaluation of healthcare practices, ethics and medical liability. Third cycle/Residency (3–6 years depending on the specialty): acquisition of knowledge and skills enabling provision of quality care in the specialty, focus on the needs of patients.	Pre-clinical years (2 years): general training in medical sciences. Clinical years (2 years): clinical rotations, receiving basic instruction and hands-on experience with patients in the major medical specialties. Residency (3 to 7 years depending on the specialty): acquisition of knowledge and skills enabling provision quality care in the specialty, focus on the needs of patients.
Presence of Nutrition Instruction	Taught in most medical schools. Only taught during pre-clinical years. Can be taught during residency depending on the specialty; there is no residency in nutrition.	Not taught in every medical school. Mostly taught during pre-clinical years and sometimes during clinical years. Can be taught during residency depending on the specialty. There is no residency in nutrition.
Format of Nutrition Instruction	Physiological and biochemical approaches to nutrients. Compulsory for all students when taught. Not taught in every medical school. Only taught during pre-clinical years.	Physiological and biochemical approaches to nutrients which are often incorporated within endocrinology class. Mostly non-compulsory. Culinary medicine programs (Teaching Kitchen, Health Meets Food, others).
Nutrition Evaluation	Nutrition is part of the national board exam whether or not medical students have been taught it in class. Mostly tested with Endocrinology.	Nutrition is not compulsory most of the time and not always evaluated.

## Data Availability

The datasets and/or references used and/or analyzed during the current study are available from the corresponding author upon reasonable request.
